# Whole Genome Sequencing and Root Colonization Studies Reveal Novel Insights in the Biocontrol Potential and Growth Promotion by *Bacillus subtilis* MBI 600 on Cucumber

**DOI:** 10.3389/fmicb.2020.600393

**Published:** 2021-01-12

**Authors:** Anastasios Samaras, Marios Nikolaidis, Maria Luisa Antequera-Gómez, Jesus Cámara-Almirón, Diego Romero, Thomas Moschakis, Grigoris D. Amoutzias, Georgios S. Karaoglanidis

**Affiliations:** ^1^Laboratory of Plant Pathology, Faculty of Agriculture, Forestry and Natural Environment, School of Agriculture, Aristotle University of Thessaloniki, Thessaloniki, Greece; ^2^Bioinformatics Laboratory, Department of Biochemistry and Biotechnology, University of Thessaly, Larisa, Greece; ^3^Instituto de Hortofruticultura Subtropical y Mediterránea “La Mayora”—Departamento de Microbiología, Universidad de Málaga, Málaga, Spain; ^4^Laboratory of Dairy Science and Technology, Department of Food Science and Technology, Faculty of Agriculture, Forestry and Natural Environment, School of Agriculture, Aristotle University of Thessaloniki, Thessaloniki, Greece

**Keywords:** *Bacillus* spp., *Fusarium oxysporum* f.sp. *radicis cucumerinum*, yellow fluorescence protein-tagging, plant growth Promoting bacteria, root colonization, whole genome analysis

## Abstract

*Bacillus* spp. MBI 600 is a gram-positive bacterium and is characterized as a PGPR strain involved in plant growth promotion and control of various plant pathogens which has recently been introduced into the agricultural practice. In this study we performed a Next Generation Sequencing analysis, to analyze the full genome of this microorganism and to characterize it taxonomically. Results showed that MBI 600 strain was phylogenetically close to other *Bacillus* spp. strains used as biocontrol agents and identified as *B. subtilis*. GOG analysis showed clusters contributed to secondary metabolites production such as fengycin and surfactin. In addition, various genes which annotated according to other plant-associated strains, showed that play a main role in nutrient availability from soil. The root colonization ability of MBI 600 strain was analyzed *in vivo* with a yellow fluorescence protein (*yfp*) tag. Confocal laser scanning microscopy of cucumber roots treated with *yfp*-tagged MBI 600 cells, revealed that the strain exhibits a strong colonization ability of cucumber roots, although it is affected significantly by the growth substrate of the roots. *In vitro* and *in planta* experiments with MBI 600 strain and *F. oxysporum* f.sp. *radicis cucumerinum* and *P. aphanidernatum*, showed a high control ability against these soilborne pathogens. Overall, our study demonstrates the effectiveness of MBI 600 in plant growth promotion and antagonism against different pathogens, highlighting the use of this microorganism as a biocontrol agent.

## Introduction

During the last decades, the use of beneficial bacteria for the biological control of plant pathogens became a major weapon in the protection of several crops, with a continuously increasing number of them registered throughout the world as biopesticides. Among those beneficial bacteria, species that have the ability to colonize plant roots and support plant growth and/or protection against pathogens are commonly referred to as plant-growth-promoting rhizobacteria (PGPR). The interactions of PGPR with plants and plant pathogens is accomplished through multiple direct and indirect modes of action. Direct mechanisms include nitrogen fixation, siderophore and phytohormone production, competition with microorganisms in rhizosphere or production of secondary metabolites (Shafi et al., 2017; Backer et al., 2018). Indirect mechanisms include the induction of systemic resistance (ISR) and the inhibition of plant ethylene synthesis (Chen et al., 2006; Doornbos et al., 2012). Within the group of PGPR, *Bacillus* spp. has a dominant role, with a continuously increasing number of strains being evaluated and used as biofertilizers or biopesticides in different crops and against a variety of soil-borne and foliar pathogens. The extensive development and registration of *Bacillus-*based products is related to some unique characteristics of the genus that include high replication rate, resistance to adverse environmental conditions, increased efficiency in plant growth promotion and broad spectrum activity (Wang et al., 2013; Magno-Perez-Bryan et al., 2015).

*B. subtilis* MBI 600 (thereafter MBI 600) is a biological control agent (BCA) commercialized recently by BASF. Currently, published information on its biocontrol and plant growth promotion efficiency is restricted on crops such as rice and tomato. Previous research conducted in our laboratory showed that MBI600 can provide high control efficacy against a major soil-borne fungal pathogen of tomato, *Fusarium oxysporum* f.sp. *radicis lycopersici* (Samaras et al., 2018). In the same host, it has been shown to be effective against important tomato viruses such as TSWV and PVY (Beris et al., 2018). More recently, gene expression studies on MBI 600-treated tomato plants revealed that this protective function against viruses is achieved through eliciting defense responses by activation of salicylic acid (SA)-responsive genes and a synergistic cross-talk between jasmonic acid/ethylene (JA/ET) and SA-signaling (Dimopoulou et al., 2019). Similarly, on rice, MBI 600 was found to be effective against *Rhizoctonia solani*, an important soil-borne pathogen of rice (Kumar et al., 2012).

Cucumber, (*Cucumis sativus* L.) is one of the most important vegetable crops cultivated throughout the world either in open fields or in greenhouses and suffers attacks from several foliar and soil-borne fungal pathogens (Keinath et al., 2018). Among the soilborne pathogens that attack cucumber plants, *Pythium aphanidermatum* causes damping off disease on young plants, whereas, *Fusarium oxysporum* f. sp. *radicis-cucumerinum* (*Forc*) causes Fusarium crown and root rot (FCRR). In the past, the most popular control methods for those diseases was either the use of fungicides with specific action against Oomycetes including *Pythium* spp. or soil fumigation with methyl bromide, that was effective against both pathogens (Pavlou and Vakalounakis, 2005). However, social concerns for the use of synthetic fungicides along with the removal of methyl bromide from the market led in an increased interest for application of BCAs in cucumber crops. During the last two decades several *Bacillus* spp. strains such as *B. subtilis* ME488, *B. subtilis* SQR-9 and *Paenobacillus polymyxa* SQR-21, have been reported to effectively control *Pythium* spp. and *Forc* in cucumber. In several cases, the control efficacy achieved by *Bacillus* strains was associated to the production of antifungal compounds and volatile organic compounds (VOCs) that induced plant defense reactions (Chung et al., 2008; Cao et al., 2012; Khalaf and Raizada, 2018).

The first whole genome sequence of a *Bacillus* strain became available for *B. subtilis* strain Marbug 168 and since then, about 200 *Bacillus* strains have been sequenced (Kunst et al., 1997; Liu et al., 2012; Franco-Sierra et al., 2020). Whole genome analysis of Bacillus species constitutes the basis to understand the interactions with plants and other microorganisms (Magno-Perez-Bryan et al., 2015; Shaligram et al., 2016). However, the precise taxonomic position of MBI 600 is puzzling since it is referred either as *B. subtilis* or *B. amyloliquefaciens*. *B. amyloliquefaciens* has been delineated from *Bacillus subtilis* (*Bs*) sensu lato based on phylogenetic differences and physiological characteristics associated with antibiotic production and root colonization ability and currently comprises of two subspecies, the plant-associated *B. amyloliquefaciens* subsp. *plantarum* and the non-plant associated *B. amyloliquefaciens* subsp. *amyloliquefaciens* (Borriss et al., 2011; Zhang et al., 2016).

Variations that have been observed in plant growth promoting capacity and biocontrol efficiency of several PGPR strains have been correlated to differences in their root-colonizing ability (Poonguzhali et al., 2008; Cao et al., 2011; Posada et al., 2018). Root colonization ability is a crucial factor for plant-PGPR interactions, determining the success of a PGPR strain in promoting plant growth and providing protection against pathogens (Kamilova et al., 2005; Ugoji et al., 2006). PGPR strains belonging to the *Bacillus* taxa are often formulated in the form of spores that are tolerant to adverse environmental conditions and the first step in growth promotion and biological control processes mediated by Bacillus applications is the spore germination. However, successful colonization of a plant root by a PGPR passes through additional stages that include attraction to the roots and establishment on them (De Souza et al., 2015; Posada et al., 2018). Numerous studies in the past have focused on the investigation of parameters affecting the colonization performance of several *Bacillus* strains (Rudrappa et al., 2008; Cao et al., 2011; Fan et al., 2011; Zhang et al., 2014; Yuan et al., 2015). Establishment of PGPR on plant roots as a stage of the colonization process is mediated through biofilm formation, surfactin production and metabolic enzymes production that are regulated by quorum sensing (Beauregard et al., 2013).

Despite the fact that root colonization ability has been extensively studied in Gram-negative bacterial strains, the number of similar studies for Gram-positive PGPR is limited because of the absence of a reliable and stably expressed molecular marker (Jansson, 2003; Cao et al., 2011). *In situ* visualization of bacterial cells on the root surface and the rhizosphere using green fluorescent protein (GFP) as a marker became a tool that has revolutionized root colonization studies by PGPR. However, the structural instability of the plasmid-based GFP vectors in Gram-positive strains limited their applications in studies aiming to determine the colonization ability of *Bacillus* spp. (Cao et al., 2011; Posada et al., 2018). Nevertheless, optimization of *Bacillus* transformation protocols along with the use of electron confocal microscopy or fluorescent *in situ* hybridization contributed to a recent increase of studies aiming to determine the root colonizing ability of several *Bacillus* strains in natural environment and different substrates (Romero et al., 2006; Fan et al., 2011; Posada et al., 2018).

Based on the above, the current study was initiated aiming to provide insights: (a) on the whole genome sequence of MBI 600 that will define the precise taxonomic position of MBI 600 and will lead to identification of genes likely to be involved in plant growth promotion and plant defense mechanisms, (b) on the ability of the MBI 600 to colonize cucumber roots using YFP-labeled bacterial cells by combining a natural transformation system and confocal laser scanning microscopy (c) on the MBI 600 ability to colonize cucumber roots grown in different growth substrates by taking advantage of the chloramphenicol resistant cassetteinserted in the *yfp*-plasmid and (d) on the MBI 600 biocontrol efficiency against two major soil-borne pathogens of cucumber, *Forc* and *P. aphanidermatum*.

## Materials and Methods

### Maintenance of MBI 600 and Plant Pathogen Strains

The MBI 600 strain used in the study was isolated from a commercial formulation of the product (Serifel 9.9 WP) kindly provided by BASF Hellas S.A. For the isolation, 1 g of product’s powder was diluted in 50 ml of dd H_2_O, centrifuged for 5 min at 4,000 rpm followed by discard of the supernatant and resuspension of the pellet in 5 ml of PBS buffer. Then, 10-fold serial dilutions were performed and 100 μl of each dilution were spread on Tryptone Soy Agar medium (TSA, LabM, Hungary) and incubated at 37°C for 24 h. 16S rRNA gene sequence analysis was used to confirm the identity of the isolated bacterial strain by using primers 27F 5′-AGAGTTTGATCMTGGCTCAG-3′) and 511R (5′-GCGGCTGCTGGCACRKAGT-3′) (Liu et al., 2015).

*Forc* and *P. aphanidermatum* isolates used in the study belong to the fungal collection of the Lab of Plant Pathology, AUTH. Both pathogens were isolated from diseased cucumber plants. The fungal isolates were grown and maintained on Potato Dextrose Agar (PDA, LabM, Hungary) slants at 4°C.

### Whole Genome Sequence and Bioinformatics

MBI 600 was cultured in 0.1% Luria Broth (LB) medium under optimal growth conditions (pH 7.0 and 37°C) for 12 h. Bacterial cells were collected by centrifugation and DNA was purified using a Qiagen Dneasy kit according to the manufacturer (Qiagen, Germany). DNA was firstly sequenced in a Pacific Biosciences platform using SMRT cell 8 Pac V3, DNA Polymerase Binding Kit P6 v2. To increase the quality of the sequenced genome. DNA was additionally sequenced with Illumina HiSeq X, using the TrueSeq DNA PCR Free (350) library kit for 150 nt paired-end reads.

The raw PACBIO subreads data files were converted to fastq files and were assembled with Blasr (Chaisson and Tesler, 2012) and Canu V.1.6 (Koren et al., 2017). The resulting assembly comprised of one circular contig. Next, the Illumina reads were assembled into contigs with Spades version 3.12 (Antipov et al., 2016), using the PacBio draft genome as trusted-contig. These contigs were subsequently used as BLASTn query against the PacBio draft genome with *e*-value cutoff of 1e-10. Four contigs were then selected that had a total of 99.2% genome coverage and were used to manually apply corrections in the PacBio genome. As a quality control, the MBI 600 genome was compared with known genomes from NCBI non-redundant nucleotide database with the BlastN web interface. The top hit was *B. subtillis* 3NA (CP010314.1) with a query coverage of 99%. Blast2Seq and the resulting dot plot clearly showed that these two genomes are co-linear except for a segment of 3 NA between positions 2.2–2.3 Mb that is missing in MBI 600. Gene annotation was performed by the NCBI Prokaryotic Genome Annotation Pipeline (Tatusova et al., 2016).

To ensure the exact phylogenetic position of this strain within the *Bacillus* genus, a phylogenomic analysis was performed with 147 *Bacillus subtilis* sensu lato proteomes, including strains from *B. subtilis, B. atrophaeus, B, amyloliquefaciens, B. velezensis, B. licheniformis, and B. paralicheniformis*. The MBI 600 strain was used as a reference point for best reciprocal Blastp hits against the other 147 proteomes (*e*-value cut off 1e-5). This resulted in 2,317 core proteins of the strain MBI 600, that were present in all other 147 proteomes and were used for the phylogenomic analysis. Each of these orthologous groups were aligned with the Muscle software (Edgar, 2004) within Seaview V.4 (Gouy et al., 2010) and were subsequently concatenated to one protein super-alignment. The super-alignment was then filtered with Gblocks default parameters (Castresana, 2000) and the resulting alignment (294,136 variable sites) was used to compute a Neighbor Joining tree with 500 bootstrap replicates and Kimura model which is embedded into the Seaview program (Gouy et al., 2010). The tree was annotated and visualized using the iTOL webserver and Treedyn software (Chevenet et al., 2006; Letunic and Bork, 2016). A second pylogenomic analysis was prepared that included MBI600 and another 69 annotated *B. subtilis* proteomes, resulting in 2,736 core proteins. These core proteins were processed as described earlier resulting in 270,725 variable sites and second phylogenomic tree was also prepared.

COG annotation was performed with the WebMGA server (Wu et al., 2011) for MBI 600, 12 *B. subtilis*, 3 *B. amyloliquefaciens*, and 1 *B. velezensis* proteome of the dataset, that are known to promote plant growth. To access the unique gene content of MBI 600 strain against other plant-associated *Bacillus* strains, BlastN was performed against 18 strains. Prediction of genes involved in secondary metabolites was conducted by antiSMASH software tool (Blin et al., 2019).

### MBI 600 Transformation and Confocoal Microscopy

*B. subtilis* MBI 600 was transformed with the strong constitutive promoter *upp* from the type strain of *Bacillus cereus* ATCC 14,579 (Eijlander and Kuipers, 2013) fused to a yellow fluorescent protein (*yfp*) inserted in the replicative plasmid pHCMC0_2_ (Nguyen et al., 2005; Caro-Astorga et al., 2020). MBI600 was transformed by natural genetic competence using protocols similar to those used for *Bacillus subtilis* strain 168. Briefly, a MBI 600 culture in 20 ml of LB medium was incubated overnight at 37°C, under continuous shaking, in a 125 ml flask. Ten ml of the overnight culture were transferred into a 15 ml falcon and centrifuged at 7,000 rpm for 7 min. The supernatant was removed and the pellet re-suspended in 10 ml of competence medium: 100 mM potassium phosphate buffer pH 7.2, 2% D-glucose, 0.01% casaminoacids, 0.02% L-glutamate (monopotassium salt), 3 mM sodium citrate and 0.022 mg/ml ferric ammonium citrate, supplemented with 3.33 mM of MgSO_4_ and 0.05 mg/ml of phenylalanine and tryptophan. The Optical Density (OD) was calculated and measured at ∼5. Cells were transferred in a 125 ml flask and transformation buffer was added until the OD was measured at ∼1. The flask was placed at 37°C into a shaker to an A_600_ 1-1, 5 (5–7 h). One ml of the culture was transferred in a sterile 1.5 ml Eppendorf tube, followed by the addition of 10 μg of the plasmid and incubation at 37°C for 45 min. In LB plates amended with 5 ng ml^–1^ chloramphenicol, 300 μl of the suspension were spread, while the rest of the suspension was centrifuged for 5 min at 4,000 × g. The remaining pellet was re-suspended in 100 μl of CM buffer and spread again in antibiotic-amended LB plates. The dishes were incubated at 37°C, overnight. Positive colonies were checked by fluorescence microscope and by colony PCR. For confocal microscopic observations of colonization ability a slightly modified procedure described previously was followed (Cao et al., 2011). Briefly, cucumber seeds were grown in a hydroponic floating system for 5 days. Then the roots were submerged in a cell suspension (OD∼0.8) of the GFP-tagged MBI 600, for 20 min and placed again in the floating system. For control plants the same procedure was applied, however, MBI 600 wild-type cells were used. Roots of cucumber plants were collected at 4, 24, and 48 h after bacterial application and washed with PBS buffer. Each root was placed on a glass slide with phosphate buffered saline (PBS, pH 7.2) under a coverslip. Observation was performed with a Leica TCS SP5 II confocal laser scanning microscope (CLSM), mounted on a Leica Model DMI 6000B inverted microscope, and operated in the fluorescence mode with a 60 × oil-immersion objective of numerical aperture 1.40. Fluorescence from the sample was excited with the 488 nm of an argon (Ar) laser line and with the 633 nm of a red HeNe laser line. The size of the images was adjusted to 512 × 512 pixels in x-y plane. The signal from the samples was collected and eight (8) scans were averaged during the creation of each image. The temperature during the microscopy tests was kept constant at 20°C.

### Colonization Assays in Various Growth Substrates

Colonization patterns of MBI 600 on cucumber roots were tested in four different growth systems: sterile conditions (gnotobiotic system), commercial peat mixture, natural soil suitable for vegetable production (vegetable soil) and hydroponic cubes (Grodan, Netherlands). In all the experimental procedures the chloramphenicol-resistant and *yfp*-labeled strain was used.

Cucumber seeds were sterilized by immersion in 1% (v/v) sodium hypochlorite for 1 min, rinsed five times in sterile water before sowing and then placed under gnotobiotic conditions in glass tubes (200 × 25 mm diameter) filled with 20 cm^3^ Perloflor^®^ and 30 g pure sea sand, mixed with 10% (v/v) nutrient solution PNS (plant nutrition solution) (Hoffland et al., 1989). After sterilization each cucumber seed was placed in the substrate. The substrate was then, drenched with 2 ml of bacterial water suspension (OD∼0.8), as described above. A negative control with distilled water was also included in the experimental design. After inoculation, glass tubes were placed in a growth chamber (16 h photoperiod and a light/dark temperature regime of 18:25°C). Roots were collected 5, 15, and 20 days after sowing. Each root was placed into a tube with phosphate buffered saline (PBS) and transferred in Elmasonic S30 to detach bacterial cells from the roots using ultrasonic waves at a frequency of 37 kHz. After appropriate dilution, the suspensions were plated onto LB plates amended with 5 ng ml^–1^ chloramphenicol. After 24 h of incubation at 37°C, colonies were counted and the concentration was calculated as cfu/ml.

For root colonization in soil environments, 14 days-old cucumber seedlings were used. Seedlings were inoculated by soaking their roots for 20 min in a suspension containing 10^7^ cfu ml^–1^ of chloramphenicol-resistant/*YFP-*tagged MBI 600. Roots treated with ddH_2_O were used as control. Plants were transferred to pots with 250 gr of 2 different types of soil, a commercial peat mixture and a vegetable soil. The commercial peat mixture contained peat moss (60%), vermiculite (15%), perlite (10%), geolite (5%), guano (2%), and humic acid (1%). In the natural vegetable soil, an analysis was conducted revealing that it was a loamy sand (pH 6–4, 0–4% organic matter (OM), 84% sand (S), 8% silt (Si), and 8% clay (C). Plants grown in these soil substrates were kept in greenhouse conditions for 20 days. For root colonization in hydroponic systems, the same procedure for the bacterial application was followed. Plants were transferred to hydroponic cubes and placed in floating systems with the appropriate nutrient solution. Samplings were conducted at three time points 5, 15, and 20 days after the application. Bacterial colonies were measured as described previously.

### Plants Growth Promotion Assays

The effect of MBI 600 on cucumber plants (cv. Bamboo) growth was estimated by measuring the following growth parameters: shoot height, root length, shoot fresh weight, and root fresh weight. Cucumber seeds were individually sown in plastic pots containing 80 cm^3^ of a 5:1 mixture of peat and perlite. Bacterial cultures were prepared in LB medium containing flasks and shaking overnight at 37°C. The suspension was then centrifuged at 4,000 × g for 5 min and the pellet was re-suspended in dd H_2_0, until the OD (measured at 600 nm) of the culture reached values of 0.8. Ten milliliter of the bacterial suspension were applied in each pot, just after sowing, by soil drenching, while the application was repeated 20 days after sowing. In addition to MBI 600 the commercially available *Bacillus amyloliquefaciens* QST713 strain (Serenade ASO, 1.34SC, BAYER CropScience), was included in the experimental design as a reference biological treatment. Seedlings were kept under greenhouse conditions. Plants were irrigated regularly but did not receive any fertilization, and watered every 2 days. Measurements were conducted 30 days after sowing. In total, there were five replicates of 10 plants each, in a complete randomized block design.

### *In vitro* Assays for the Antagonistic Activity of MBI 600 Against *Forc* and *P. aphanidermatum*

MBI 600 was tested for its ability to inhibit the growth of *Forc* and *P. aphanidermatum* in dual-cultures (Romero et al., 2004). Dual cultures consisted of the bacterial isolate and each of the 2 fungal isolates inoculated on opposite sides of the plate at approximately 10 mm distance from the margins of the plate. The bacterial cells were streaked as a straight line onto PDA medium in 9-cm diameter Petri dishes and the plates were inoculated with a 6 mm-diameter plug of mycelium taken from the colony margins of actively growing 7 day-old cultures. Plates were incubated for 7 days at 25°C and antagonistic activity was evaluated by measuring the diameter of the fungal colonies and the length of the inhibition zones (mm). Five replicate dishes were prepared per treatment and the experiment was repeated 3 times.

### Assays for the MBI600 Biocontrol Efficiency of Against *Forc* and *P. aphanidermatum* on Cucumber Plants

Eleven days-old cucumber plants (cv. Bamboo) were inoculated with *Forc* and *P. aphanidermatum*. For the production of *Forc* inoculum, mycelium was placed on PDA in 9 cm Petri dishes and incubated at 25°C for 7 days in darkness. Four mycelial plugs, taken from 7 day-old cultures, were transferred into 250 mL Czapek-Dox broth (CDB; Duchefa, Haarlem, The Netherlands) in 500 mL Erlenmeyer flasks and incubated for 3 days at 28°C in a rotary shaker at 150 rpm. After filtration through 4 layers of cheesecloth, the concentration of the resulting spore suspension was estimated by using a haemocytometer under light microscopy and adjusted to 5 × 10^5^ conidia ml^–1^. For *P. aphanidernatum*, inoculum was prepared in V-8 liquid medium (200 ml of vegetable juice V8 and 3 g of CaCO_3_ per L of deionized water). Flasks were incubated for 10 days at 25°C in the dark without shaking, Then, mycelial mats were washed two times with tap water and blended for 30 s at high speed in a blender (Waring, New Hartford, United States). Oospores were counted with a haemocytometer and their concentration was adjusted at 7 × 10^3^ ml^–1^.

For the artificial inoculation of the plants, each pot was drenched with 10 ml of the inoculum suspensions. Control plants were drenched with sterile distilled water. The application of MBI 600 was conducted by drenching each pot with 15 ml of bacterial suspension (OD ∼0.8), 24 h before the inoculation with the pathogens. In addition to MBI 600, in the experimental design a standard chemical and a standard biological reference treatment were included. *Ba* QST713, (Serenade ASO, 1.34SC, BAYER CropScience, Greece) was the biological reference treatment applied at the commercially recommended dose of 16 ml L^–1^, f. p., 24 h before the inoculation of the plants with the pathogens. 8-hydroxyquinoline (Beltanol 37.5 SL, Agrology SA, Greece) was the chemical reference treatment applied at the commercially recommended dose of 0.53 ml L^–1^, f.p. 24 h before the inoculation of the plants.

FCRR symptoms were assessed 10 days after the inoculation of the plants using a 0–4 disease scale (Chen et al., 2010). Damping off symptoms caused by *P. aphanidernatum* were assessed using a 0–1 disease index scale, 7 days after the inoculation of the plants. Fifty cucumber plants per treatment were inoculated and the experiment was repeated 3 times.

### Statistical Analyses

Data of the independent replications on plant growth parameters, disease incidence/severity and bacterial cell enumeration in colonization experiments, were combined after testing for homogeneity of variance using Levene’s test. The combined data were then, subjected to one-way analysis of variance (ANOVA). Duncan’s Multiple Range Test was used for comparison of means. The statistical analysis was supported by SPSS 21.0 (SPSS, Chicago, IL, United States).

## Results

### Phylogenomic Analysis of *B. subtilis* MBI 600

**FIGURE 1:**
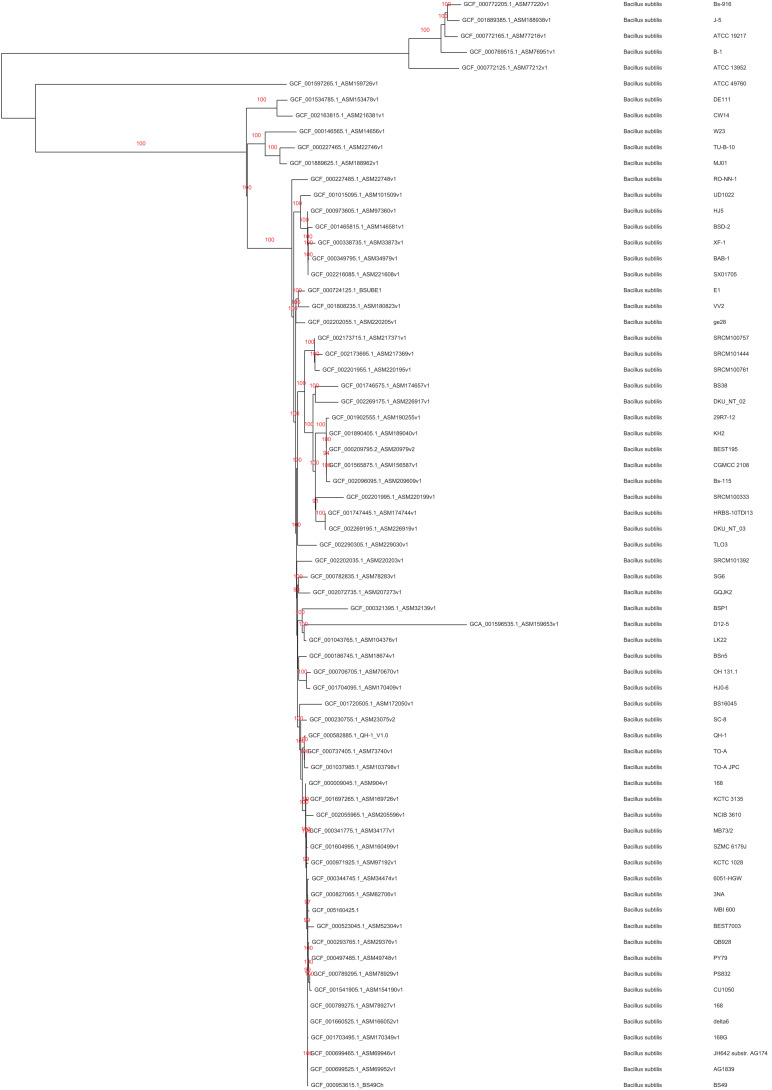
Phylogenomic Neighbor Joining tree (with Kimura two parameter model and 500 bootstraps) of 70 *Bacillus subtilis* proteomes based on 2,736 core protein orthologous groups.

**FIGURE 2:**
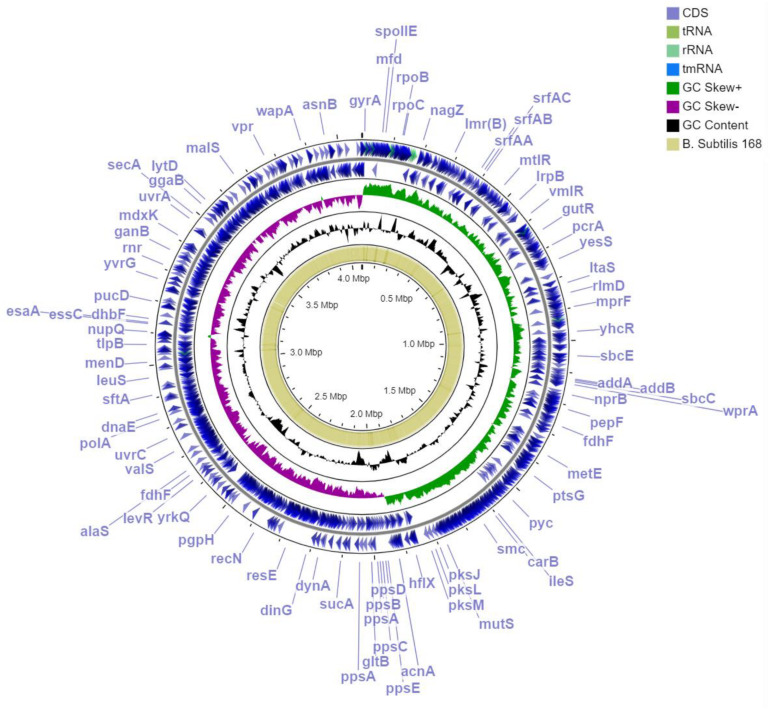
Circular representation of *Bacillus subtilis* MBI 600 genome for several specific genome features. Outermost, 1st and 2nd annotated genes (blue); 3rd GC skew+ (green); 4th GC skew- (violet); 5th GC content (black); blast orthologs of B. subtilis str.168. Visualization was performed by using GC viewer server V1.0.

To define the MBI 600 taxonomy, gene analysis of the 16sRNA was initially performed. BLAST analysis showed that the bacterial strain under investigation belongs to the *B. subtilis* species with a high coverage (100%) and identity (99%) score. To further reveal the evolutionary relationship of MBI 600 with other *Bacillus* spp. strains, a phylogenomic analysis was performed including 2,317 and 2,736 core proteins of 2 different sets of organisms, *B subtilis sensu lato* and *B. subtilis* species, respectively. The first phylogenomic tree clearly showed that MBI 600 is a member of the *Bacillus subtilis* subsp. *subtilis* evolutionary group and not a member of the *B. amyloliquefaciens* evolutionary group (Supplementary Figure 1). The second phylogenomic tree revealed the position of MBI 600 within the *B. subtilis* species (Figure 1). Compared to the closest plant associated genome of *B. subtilis* str. 168, which is already characterized as a biocontrol agent, the genome of MBI 600 was found to share identity higher than 95% (Figure 2).

### Genome Analysis of *B. subtilis* MBI 600

**FIGURE 3:**
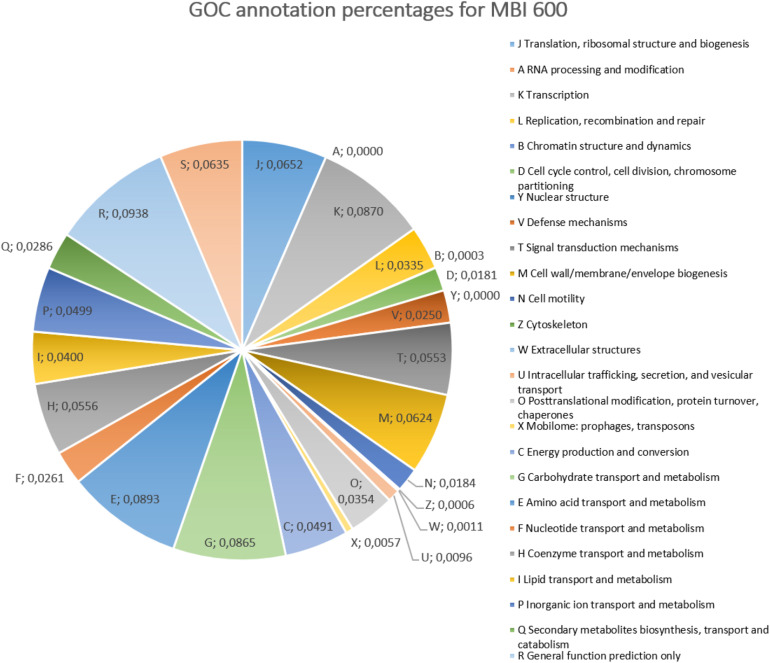
Pie chart of GO (Gene Ontology) analysis summarized *Bacillus subtilis* MBI 600 genes, according to molecular function.

Upon genome sequencing and assembly, MBI 600 was given as circular chromosome of 4,076,736 bp with a GC content of 43.84%, a size similar to that of other *Bacillus* genomes and was deposited in Genbank (Accession Number CP033205.1). The genome annotation report by NCBI revealed that the genome constituted of a total of 4,259 genes, with 4,076 coding CDS, 121 RNA genes, 86 tRNAs (Figure 2). After annotation, 2,850 CDSs were assigned to putative biological functions whereas 1,229 CDSs were characterized as hypothetical proteins with unknown function. Using Gene Ontology Consortium (GOC) analysis, 25 functional classes were identified with some related to biocontrol activity, such as production of secondary metabolites. In addition, some of the functional classes identified by the GOC analysis were related to biofertilizing, such as inorganic ion transport and metabolism (Figure 3).

**FIGURE 4:**
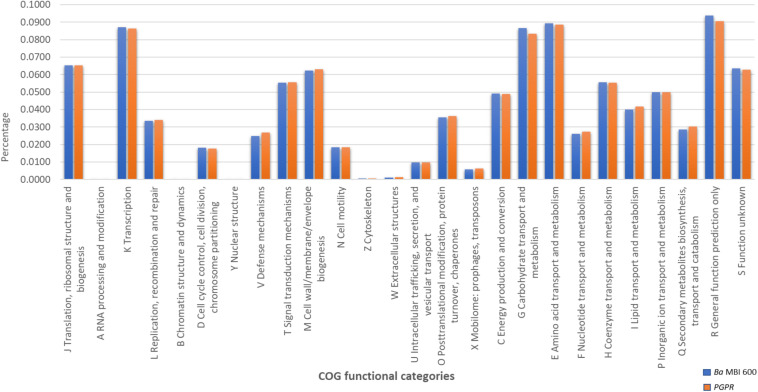
GOC annotation differences between *Bacillus subtilis* MBI 600 and 16 additional Plant Growth Promoting *Bacillus* sp. strains.

A targeted GOC analysis was further conducted to compare the MBI 600 genome to the genomes of 18 different plant-associated strains belonging to the *Bacillus* cluster. This analysis revealed that the number of MBI 600 annotated genes was almost equal to the other strains for the majority of the functional categories. Further molecular evidence for the PGPR properties of the MBI 600 strain was provided by the GOC comparison with above mentioned PGPR *Bacillus* strains which revealed that the GOC content of MBI 600 was remarkably similar to that of the 18 reference PGPR isolates (Figure 4). A comparative analysis was further performed by focusing on 3 well-known biocontrol agents, *B. subtilis* 168, *B. amyloliquefaciens* FZB42 and *B. amyloliquefaciens* UMAF 6,639. Most genes related to plant growth promotion and plant protection were detected in all PGPR strains including MBI 600 (Table 1).

**TABLE 1:** Presence (+) or absence (−) of selected genes associated with plant growth promotion and plant protection against pathogens in biocontrol agents belonging in the Bacillus family.

Gene	Annotation	Function	PGPR species^a^			
			*Bs MBI 600*	*Bs 168*	*Ba FZB 42*	*Ba UMAF 6,639*
*yvra*	Iron ABC transporter ATP-binding protein	Putative iron availability	+	+	+	+
*yvrb*	Corrinoid ABC transporter permease	Putative iron availability	+	+	+	+
*yvrc*	Corrinoid ABC transporter permease	Putative iron availability	+	+	+	+
*Nar*	Nitrate trasporter	Nitrate transporter	+	+	+	+
*Nas*	Nitrate trasporter	Nitrate transporter	+	+	+	+
*ktrA*	Potassium transporter	Potassium transporter	+	+	+	+
*yugO*	Potassium channel	Potassium transporter	+	+	+	+
*mgtE*	Magnesium transporter	Magnesium transporter	+	−	+	−
*Ktr*	Potassium uptake	Potassium transporter	+	+	+	−
*srfa*	Surfactin synthase subunit 1	Secondary metabolite production	+	+	+	+
*srfb*	Surfactin synthase subunit 2	Secondary metabolite production	+	+	+	+
*srfc*	Surfactin synthase subunit 3	Secondary metabolite production	+	+	+	+
*sfp*	Fengysin production	Secondary metabolite production	+	−	+	−
*npr*	Bacillolysin	Secondary metabolite production	+	+	−	−
*sbo-alb*	Antilisterial bacteriocin subtilosin biosynthesis protein AlbB	Secondary metabolite production	+	+	−	−
*flgB*	Flagellar coding protein	Cells active movevent	+	−	+	+
*fliD*	*Flagellar coding protein*	*Cells active movevent*	+	−	+	+

*^*a*^Bs, Bacillus subtilis; Ba, Bacillus amyloliquefaciens.*

(i) Growth promotion

Beneficial bacteria contribute to plant growth promotion by involving into nutrients uptake and different unique genes are related with each of the specific nutrient elements. Two gene clusters, *nas*A-*nas*B-*nas*C and *nar* (H-Z-J-I-G) were found and annotated as nitrate transporter and as nitrate reductase, respectively, and predicted to be involved in nitrate transport and reduction. Two genes for magnesium transportation, *mgt*E and *yqx*L, were predicted to a double function, uptake of nutrients and detoxification of heavy metals ions for the host plant and the bacteria. In addition a gene cluster consisting from 4 genes *ktr* (A–D) was found in MBI 600 genome and is predicted to be involved in potassium uptake (Table 1).

(ii) Root colonization

Flagellar proteins play major role in the colonization ability of PGPR strains. In the MBI 600 genome we found in total 36 genes involved in flagellar protein coding. The majority of these genes was localized in 2 clusters, the flg cluster consisted of 5 genes (*flg*B—*flg*C—*flg*E—*flg*K—*flg*M) and a larger cluster with 16 genes (*fli*D- *fli*E- *fli*F- *fli*G- *fli*H- *fli*IJ- *fli*K- *fli*L- *fli*M- *fli*P- *fli*Q- *fli*S- *fli*T- *fli*Y- *fli*Z) (Table 1).

(iii) Direct inhibition–antibiotic production

PGPR strains produce a variety of antibiotics involved in the direct inhibition of plant pathogens in the root vicinity. Sequence of *srfA*, *srfB*, and *srfC* genes, that are likely involved in surfactin synthesis, the respective sequences of the FZB42 was highly similar to the MBI600 strain. In addition, the presence of the fengycin biosynthesis associated gene cluster consisting of 5 NRPs (ppsA-ppsE), was also found in MBI 600, with a sequence highly similar to that of the respective genes of FZB24. In addition to the above mentioned genes that identified with high similarity to genes from other PGPR strains, the prediction analysis with antiSMASH software revealed the existence in MBI 600 genome of additional regions that, according to the prediction, were involved in other antibiotic biosynthesis including compounds such as bacillaene, bacillibactin, subtilosin A and basilysin (Table 2).

**TABLE 2:** Prediction of clusters in *Bacillus subtilis* MBI 600 genome involved in secondary metabolites production, using AntiSMASH tool.

Regions (bp)	Type	Compound	Similarity%
Region 1 (204,359–226,256)^a^	Sactipeptide	Sporulation killing factor	100
Region 2 (358,311–421,751)	NRPs	Surfactin	82
Region 3 (1,761,171–1,866,418)	TransAT-PKs	Bacillaene	100
Region 4 (1,932,857–2,015,146)	NRPs	Fengycin	100
Region 5 (3,119,403–3,169,144)	NRPs	Bacillibactin	100
Region 6 (3,686,669–3,708,280)	Sactipeptide	Subtilosin A	100
Region 7 (3,711,289–3,752,707)	Other	Basilysin	100

*^*a*^The specific nucleotide position of its region, in the full genome of Bs MBI 600, according to AntiSMASH tool.*

### Root Colonization Ability of YFP-Tagged MBI 600

**FIGURE 5:**
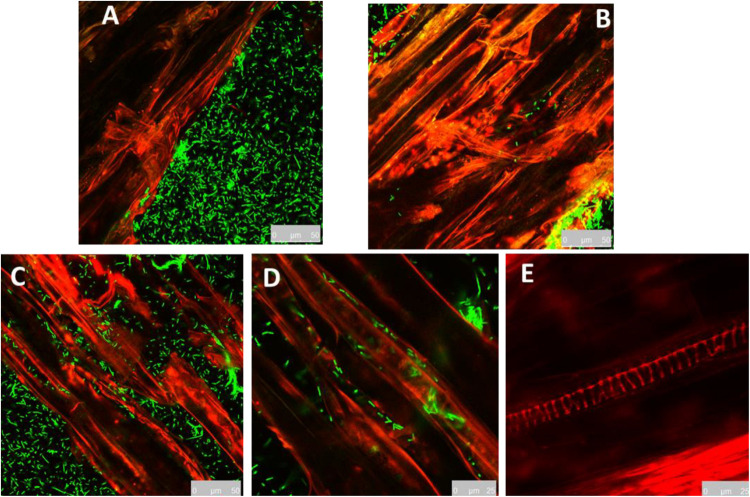
Root colonization of YFP–tagged *Bacillus subtilis* MBI 600, visualized under confocal scanning laser microscope. **(A)** 4 h post inoculation, **(B)** 24 h post inoculation, **(C,D)** 48 h post inoculation, bacterial cells in the elongation zone of cucumber root. **(E)** Cucumber root inoculated with non YFP-tagged bacterial cells.

In order to realize the colonization patterns of MBI in the cucumber root surface, cucumber roots were inoculated with YFP-tagged bacterial cells and observed by confocal microscopy. In addition, the required colonization time was investigated. The MBI 600 YFP-tagged cells emitted a constant fluorescence allowing to easily distinguish them from the background root auto-fluorescence (Figure 5). In contrast, no fluorescent cells were observed in the roots of plant treated with the non YFP-tagged bacterial strain. The confocal microscopy images showed that colonization of the root surface initiated 24 h after the immersion. After 48 h, bacterial cells were clearly visible along the entire length of the root segment. Laser scanning in different internal root layers did not show any presence of YFP-tagged bacterial cells (data not shown). Cells were observed on root surface as single cells or clustered in microcolonies (Figure 5). Transformed cells of MBI 600 were tested and showed that the *yfp* gene didn’t affect growth parameters (Supplementary Figure 3).

In order to determine the ability of MBI 600 to colonize cucumber roots growing in different substrates, to determine the application availability of the strain in various cultivated methods. To achieve that we successfully constructed a yfp-labeled mutant of the strain. Bacterial suspensions obtained from 1 cm homogenized root tissues were spread on TSA plates supplemented with 10 μg ml^–1^ of chloramphenicol, in which only transformed cells and no wild type cells of MBI 600 showed growth. The counts of bacterial cells on the chloramphenicol-amended medium showed that MBI 600 strain was able to successfully colonize cucumber roots in all the 4 different growth substrates tested, although with ranging effectiveness between these different systems. In all the 4 different growth substrates tested the higher levels of bacterial presence on cucumber roots was observed at 1st sampling, 5 days post application. At this time point the higher counts of bacterial cells were measured in the gnotobiotic system and in the commercial peat mixture with values of 3 × 10^8^ cfu cm^–1^ and 3.2 × 10^7^ cfu cm^–1^, respectively (Table 3). The colonization pattern remained the same up to the 10th day, although a decrease in bacterial population was observed 15 and 20 days after inoculation in all 4 growth substrates (Table 3). Nevertheless, the gnotobiotic and the commercial peat mixture systems still recorded the greatest colonization levels. The hydroponic system proved to be the least effective concerning bacterial colonization, showing the lowest amount of bacterial population in every single time period.

**TABLE 3:** Counts (cfu cm^–1^) of chloramphenicol-resistant/YFP-tagged *Bacillus subtilis* MBI 600 strain on cucumber roots grown in 4 different growth substrates.

Growing system^a^	Days after inoculation		
	5	15	20
Gnotobiotic system	3 × 10^8^ a^b^	3.2 × 10^6^ a	3.2 × 10^5^ a
Commercial Peat mixture	3.2 × 10^7^ a	4 × 10^5^ ab	2.5 × 10^4^ a
Vegetable soil	2 × 10^5^ ab	2 × 10^4^ b	1.5 × 10^4^ a
Hydroponic cubes	4 × 10^4^ b	2 × 10^2^ c	1.7 × 10^2^ b

*^*a*^Initial application rate of Bs MBI 600 was 2 × 10^10^ cfu ml^–1^.^*b*^Mean values followed by different letters in the column indicate significant differences among treatments according to Duncan’s Multiple Range Test (P ≤ 0.05).*

### Growth Promotion of Cucumber Plants

Pot experiments with applications of MBI 600 in cucumber plants allowed to investigate the effect in plant growth promotion. Measurements of the growth parameters on cucumber plants after 35 days under greenhouse conditions revealed that applications of MBI 600 resulted in a significant (*P* < 0.05) increase in shoot height, root length and shoot fresh weight compared to that of the untreated control plants (Table 4). Differences in root length and shoot height of untreated control plants and plants treated with MBI 600 are evident in Supplementary Figure 2. Similarly, applications of the reference BCA product *Ba* QST 713 resulted in an increase (*P* < 0.05) of root length and shoot fresh weight compared to that of control treatment, while, no difference (*P* > 0.05) was observed between the control treatment and *Ba* QST713 regarding shoot height. In contrast, no significant differences were observed (*P* > 0.05) among control and biological treatments in root fresh weight (Table 4).

**TABLE 4:** Effect of *Bacillus subtilis* MBI 600 applications on cucumber plants growth parameters compared to the growth of untreated control plants and *Bacillus amyloliquefaciens* QST713-treated plants (reference biological treatment).

Treatment	Growth parameter			
	Shoot height (cm)	Root length (cm)	Shoot fresh weight (gr)	Root fresh weight (gr)
Control	13.09 a^∗^	30.10 a	15.97 a	2.78 a
*Bs* MBI 600 (OD = 0.7)	17.23 b	40.40 b	16.42 b	2.14 a
Ba QST 713 (OD = 0.7)	15.35 ab	48.50 c	15.94 a	2.17 a

*^∗^Mean values followed by different letters in the column indicate significant differences among treatments according to Duncan’s Multiple Range Test (P < 0.05).*

### *In vitro* Antagonistic Activity of MBI 600 Against *Forc* and *P. aphanidermatum*

**FIGURE 6:**
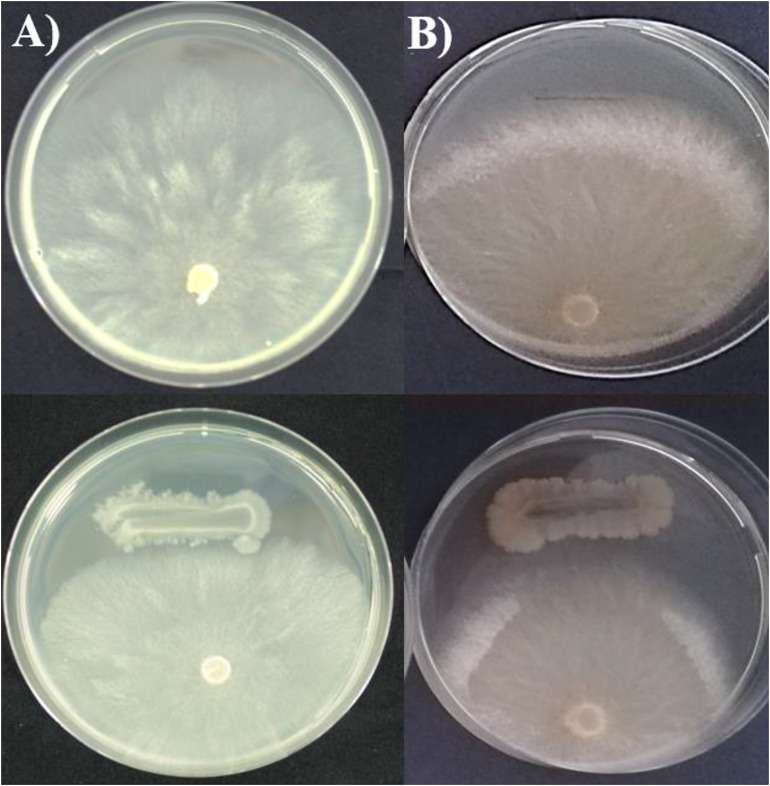
Mycelial growth of **(A)** *Pythium aphanidermatum* and **(B)** *Fusarium oxysporum* f.sp. *radicis-cucumerinum* in the absence (up) and in the presence (down) of *Bacillu subtilis* MBI 600.

The *in vitro* antagonistic activity of MBI 600 was tested on PDA, a nutrient medium suitable for the growth of all the 3 microorganisms used in the study. After 7 days of dual culturing with the plant pathogens, MBI 600 reduced significantly the mycelial growth of both *Forc* and *P. aphanidermatum*. More specifically, the relative inhibition of mycelial growth for *Forc* and *P. aphanidernatum* in the presence of MBI 600 was 26 and 33%, respectively. In addition, formation of inhibition zone of mycelial growth was observed in the dual cultures with both pathogens (Table 5 and Figure 6).

**TABLE 5:** Effect of *Bacillus subtilis* MBI 600 on the *in vitro* mycelial growth of the cucumber pathogens *Fusarium oxysporum* f.sp. *radicis-cucumerinum* and *Pythium aphanidermatum* after 7 days in dual culture.

Treatment	Pathogen					
	*Fusarium oxysporum* f.sp. *radicis cucumerinum*			*Pythium aphanidermatum*		
	Colony diameter (mm)	Relative inhibition	Inhibition zone^*a*^	Colony diameter (mm)	Relative inhibition	Inhibition zone
Control (Pathogen)	46b^*b*^	0b	**−**	60b	0b	**−**
MBI 600 + Pathogen	25a	26a	+	45a	33a	+

*^*a*^Diameter (mm) of inhibition zone between pathogens and biocontrol agent on PDA plates: **−** no inhibition, +, inhibition zone of < 10 mm; ++, inhibition zone of > 10 mm. ^*b*^Mean values followed by different letters in the column indicate significant differences among treatments according to Duncan’s Multiple Range Test (P ≤ 0.05).*

### Biocontrol Activity of MBI 600 Against *Forc* and *P. aphanidermatum*

To determine the ability of MBI 600 to control plant pathogens, we selected 2 main soil-borne pathogens of cucumber, *Pythium aphanidermatum* and *Fusarium oxysporum* f.sp. *radicis-cucumerinum.* MBI 600 significantly inhibited both *Fusarium* crown and root rot and *Pythium* damping-off on cucumber plants in greenhouse pot experiments. The applications of MBI 600 resulted in a significant reduction of disease severity compared to that observed in the untreated control treatment both for FCRR and *Pythium* damping off (Table 6). The observed control efficacy reached values of 80 and 85% for FCRR and *Pythium* damping off, respectively (Table 6). The observed control efficacy values achieved by MBI 600 applications were similar (*P* < 0.05) to that achieved by reference chemical treatment (8-hydroxyquinoline) and higher (*P* > 0.05) to that achieved by the reference biological treatment (*Ba* QST713) (Table 6).

**TABLE 6:** Biocontrol efficacy of *Bacillus subtilis* MBI 600 against Fusarium crown and root rot and *Pythium* damping off diseases on cucumber plants in pot experiments.

Treatment	Application rate	Pathogen			
		*Fusarium oxysporum* f.sp. *radicis-cucumerinum*		*Pythium aphanidermatum*	
		Disease severity^*a*^	Control efficacy (%)	Disease severity	Control efficacy (%)
*Ba* MBI600	15 ml L^–1^ (OD∼0.8)	0.09 a^*b*^	80 b^*c*^	0.02a	85 b
*Ba* QST713^*d*^	15 ml L^–1^ (OD∼0.8)	0.25 ab	73 a	0.11ab	68 a
8-Hydroxyquinoline^*d*^	0.53 ml L^–1^, f.p.	0.06 a	90 b	0.06a	90 b
Control	−	1.09 b	−	0.54b	−

*^*a*^Disease severity values for Forc and P. aphanidermatum were measured based on 0–4 and a 0–1 disease scale, respectively. Measurements were conducted 10 days after artificial inoculation of the plants.^*b*^Values followed by different letter in the column are significantly different at P = 0.05, according to Duncan’s Multiple Range Test.^*c*^Values followed by different letter in the column are significantly different at P = 0.05, according to X^2^-test.^*d*^Bacillus amyloliquefaciens QST713 (Ba QST713) and 8-hydroxyquinoline were the commercial biological and chemical reference treatments, respectively.*

## Discussion

In the current study we employed a multitasking strategy to understand the beneficial role of a recently commercialized biopesticide, *B. subtilis* MBI 600 on cucumber plants. The strain was already known for its biocontrol ability against soilborne pathogens such as *Fusarium oxysporum* f.sp. *radicis-lycopersici* on tomato plants and *Pythium* spp. on sugar beet seedlings (Schmidt et al., 2004; Samaras et al., 2018), while more recently was shown that it can exhibit antiviral action against viruses infecting tomato (Beris et al., 2018). This antiviral activity on tomato was mediated by the activation of SA-responsive genes and a synergistic cross-talk between JA/ET- and SA-signaling that triggered defense responses (Dimopoulou et al., 2019).

The whole genome sequencing and annotation of MBI 600 conducted in this study was selected as a powerful tool to determine the taxonomic position of the strain and to further study the molecular basis of mechanisms involved in plant growth promotion, root colonization and plant protection against fungal pathogens. Genomic assembly of MBI 600 was found to be similar to that of other *B. subtillis* genomes (Liu et al., 2018; Rahimi et al., 2018). The taxonomy identification between the Bacillus species is very difficult and is not clear with strict lines were each isolate belongs. In order to be more accurate, we conducted phylogenetic analysis with the 2 main species *Bacillus subtillis* and *Bacillus amyloliquefaciens.* The taxonomy identification was performed by a phylogenetic analysis that included several other plant-associated or non-associated *Bacillus* sp. Strain MBI 600 was classified in the *Bacillus subtilis* subsp. *subtilis* group, in the same branch with other plant-associated strains such as 168 and XF-1 (Guo et al., 2015).

Previous studies that compared the genome of plant- and non-plant-associated *Bacillus* spp. showed that various genes involved in biosynthesis of secondary metabolites were more abundant in plant-associated strains (Zhang et al., 2016). The GOC analysis conducted in our study to compare the genome of MBI 600 to the genomes of 18 different plant-associated strains belonging to *Bacillus* complex showed that the number of annotated genes from MBI 600 was almost equal, compared to the other strains in the majority of function categories. Such comparison provides an indirect evidence for the ability of MBI 600 to be a powerful agent of plant growth promotion and successful biological control of plant pathogens.

It is well established that enhancement of plant hormone biosynthesis that is closely related to nutrient uptake availability, mediates plant growth promotion and yield (Chen et al., 2007). Pot experiments with applications of MBI 600 in cucumber plants revealed an increase in shoot height and root length. In a previous study of our group, a similar growth pattern had been observed in tomato plants treated with MBI 600 (Samaras et al., 2018). The MBI 600 genome annotation conducted in the current study demonstrated that a large number of MBI 600 genes were involved in plant growth by enhancing nutrient up-take and availability. In detail, in MBI 600 genome, the nitrate transporters *nark* and *nas* clusters (A-B-C), the nitrate reductase narH-narZ-narJ-narI-narG and their putative regulator gene *arf*M were found. These gene clusters are predicted to be involved in nitrate transport and reduction (Wray et al., 1994). In addition to genes involved in nitrate transport, the existence of genes involved in potassium transport were identified in MBI 600 genome. Potassium is one of the most important elements in plant nutrition and PGPR play the main role for plant up taking from the soil (Hayat et al., 2010). Two genes, *ktr*A and *yug*O that had been identified and characterized as K transporters in *B. subtilis* (Holtmann et al., 2003) were also found in MBI 600 genome. In a recent full genome analysis of *B. subtillis* XF-1, some genes were found and predicted to be involved in magnesium uptake and de-toxification of heavy metal ions in host plants (Guo et al., 2015). In MBI 600 *mgt*E and *yqx*L were found and probably play the same role. Iron is an important micronutrient, which acts as a co-factor in more than 120 enzymatic activities, including chlorophyll biosynthesis and is thus, related to plant growth (Brittenham, 1994; Miller et al., 1995). The role of PGPR in iron availability is very crucial and succeeded by the siderophore production involved in the process of chelating ferric iron from the soil (Schalk et al., 2011). Strain MBI 600 is able to produce siderophores (data not shown). In addition, a cluster consisting of 3 genes (*yvr*A-*yvr*B-*yvr*C), was found to the MBI 600 genome and predicted on putative iron availability. The same cluster was reported in 2 *B. amyloliquefaciens* strains, CECT 8,237 and 8,238 (Magno-Perez-Bryan et al., 2015). In addition to the previously mentioned genes identified by the whole genome sequencing, antiSmash analysis of MBI 600 genome detected a domain/region with a very high query of bacillibactin synthesis. Bacillibactin is involved in the uptake of iron ions from the natural environment under iron limitation (Chen et al., 2009). However, the presence of this specific cluster doesn’t guarantee the production of the bacillibactin by MBI 600 as has been observed in other *Bacillus* strains such as *B. subtillis* 168 (May et al., 2001). A MALDI-TOF analysis could provide a clear evidence related to the production of bacillabactin by MBI 600. To determine the ability of MBI 600 to control plant pathogens, we selected 2 main soil-borne pathogens of cucumber, *Pythium aphanidermatum* and *Fusarium oxysporum* f.sp. *radicis-cucumerinum*. Assessment of the antagonistic activity of MBI 600 against the 2 pathogens in dual cultures *in vitro* revealed a significant reduction of mycelial growth of both pathogens. Previous studies with other *Bacillus* strains such as B068150 or SQR -9 revealed variable results against *Forc* when their antagonistic activity was tested *in vitro* (Cao et al., 2011; Li et al., 2012). Such variability is most probably related to the BCA ability to produce antifungal compounds. A lot of *Bacillus* strains are able to synthesize enzymes and non-ribosomal peptide synthetases, which are composed of multi-modulary arranged catalytic domains, catalyzing peptide formation (Stein et al., 2005). Amongst them, surfactin, fengycin, bacillomycin D, and bacillicin are the most important, indicating hemolytic, antimicrobial and antiviral activities (Chen et al., 2009). The whole genome sequence conducted in our study revealed the presence of genes encoding several of these metabolites. For instance, the gene cluster *srf* (A-B-C) that is involved in surfactin production was found in MBI 600 genome. Fengycin, a cyclic lipodecapeptide, that is highly active against filamentous fungi, was firstly identified by Nishikori et al. (1986). It is biosynthesized by a gene cluster (*pps*A-*pps*E) that was detected in MBI 600 and showed a high similarity to FZB42 cluster. In addition, the AntiSmash tool predicted a region (6) that is associated to Suntilosin A production, while two additional genes, *npr* and *sbo-alb*, found in the genome of MBI 600 are involved in the production of bacillolysin and subtolisin, respectively. These antibiotics are already known for their activity against fungal and bacterial pathogens (Halimi et al., 2010; Goswami et al., 2016). Studies on the isolation and characterization of lipopeptides with antimicrobial activity produced by MBI 600 are now being carried out in our laboratory.

In our experiments, strain MBI 600 was able to control *Fusarium* crown and root rot and *Pythium* damping off. Two drenching applications in cucumber seedlings was found to increase the control efficacy in levels equal to that of chemical treatment. Several *Bacillus* strains were reported to control these pathogens in different conditions and environments, and confirm our results (Raza et al., 2017). Our experiments underline that biocontrol agents might be an effective solution against soilborne pathogens of cucumber seedlings. Nevertheless, field experiments are necessary to verify the efficacy of MBI 600 against these pathogens and to determine the effects of natural interactions and soil conditions on its performance.

It is well established that root colonization ability plays a crucial role in the interaction between plants and PGPR (Fan et al., 2011; Vacheron et al., 2013). PGPR that are successful colonizers of plant roots reach the surface of the roots in 2 different ways, either by passive movement in water fluxes or by active flagella- propelled swimming. The active movement is determined by a special genetic motif that has been identified in most *Bacillus* sp. This motif consists of one flagella biosynthesis operon (*flalche*) and two stator elements *mot*AB and *mot*PS (Werhane et al., 2004). Two gene clusters related to flagellar motion, *flg* with 5 genes and *fli* with 16 genes were found in MBI 600 genome. Genes from these clusters were found in several *Bacillus* strains genomes and are associated with root colonization ability (Guo et al., 2015; Magno-Perez-Bryan et al., 2015).

Another major objective of our study was to determine the ability of MBI 600 to colonize cucumber roots growing in different substrates. To achieve that we successfully constructed a yfp-labeled mutant of the strain. To accomplish high stability and to avoid genetic burden, we chose to integrate a single copy of the *yfp* gene by using natural DNA transformation and take advantage of a functional homologous recombination system, that reported in a previous study for *B. amyloliquefaciens* FZB42 (Koumoutsi et al., 2004). Transformed cells of MBI 600 were tested and showed that the *yfp* gene didn’t affect growth parameters and seems to be suitable for long term studies, carried out in natural environments As we expected, the *yfp*-labeled cells were more brightly fluorescent when growing in LB media compared to cells grown on plant roots. Other studies with other *gfp*-labeled from *Bacillus* sp. showed an opposite effect suggesting that the expression levels of fluorescence protein are strain-dependent, while, in addition the root exudates of each host consisting of different metabolites may affect fluorescence level (Fan et al., 2011). The results of our study showed that the *yfp*-labeled cells of MBI 600 needed at least 24 h to colonize the primary root of cucumber seedlings. Confocal microscopy showed that bacterial cells colonize the surface of the primary root and mainly the lower rhizoplane part. This observation could be explained by the precise localization of root exudates. Previous studies with FZB42 showed that rhizobacteria colonized only a small part of rhizoplane, mainly in the region between epidermal cells and areas where lateral roots arise (Timmusk et al., 2005; Cao et al., 2011; Fan et al., 2011). Unfortunately, we couldn’t proceed to more observations and comparisons of colonization ability in more root parts since the experiment was conducted in roots of very young seedlings. Further research on roots of older plants will aid toward a more detailed localization of MBI 600 growth on plant roots. Nevertheless, images from confocal microscope showed that MBI 600 cells were localized only in rhizoplane. This pattern suggests that MBI 600 is a true epiphyte as has been previously observed for other *Bacillus* spp. commercialized as BCAs such as *B. amyloliquefaciens* FZB42 on different plant species and *B. subtilis* SQR9 on cucumber roots (Cao et al., 2011; Fan et al., 2011).

The colonization ability of MBI 600 was tested on cucumber roots grown in 4 different substrates and plate counts showed that MBI 600 had the ability to colonize the roots in all the 4 different substrates, although with marked differences in colonization efficiency. In all the 4 substrates the higher densities of the introduced strains were recovered from the rhizoplane 5 days after the introduction, while densities remained high until 15 days after BCA application. Comparisons among the 4 substrates showed the higher population densities were observed in roots grown in the gnotobiotic system and in the peat mixture. The microbial community in these 2 substrates was probably more “poor” than in the remaining 2 substrates, so the antagonism for space and nutrients was low. In addition, there are a lot of biotic and abiotic factors such as root exudates, chemical signaling between microorganisms, acidification and high molar C/N ratios that may affect colonization of roots grown in a gnotobiotic system (Dutta and Podile, 2010). The lower bacterial densities were counted in cucumber roots grown in the grodan cubes. Such low colonization ability of MBI 600 in the grodan cubes is probably related to toxicity of the mineral solution used for the nutrition of the plants. *In vitro* bioassays conducted aiming to determine the effect of the mineral solution on the growth of MBI 600 confirmed this hypothesis (data not shown). Such a toxic effect of mineral solutions used for plant nutrition on the bacterial growth has been previously observed in PGPR strains (Lee and Lee, 2015). In vegetable soil, the number of recovered bacterial cells was lower than that from roots grown in the peat mixture or the gnotobiotic system. Such finding can be explained by the ecology of bacteria competition and many mechanisms involved in that. As has been shown by studies conducted in our lab MBI 600 is a strain able to produce siderophores (unpublished data). However, production of siderophores is energy costly and therefore, siderophore-producing populations are “available” to social cheating by individuals that lose this ability but maintain the capacity to take them up (West and Buckling, 2003; Steinauer et al., 2016). This mechanism creates an energy imbalance between the microbial populations (Hibbing et al., 2010) and possibly contributes to the lower recovery of MBI 600 in the vegetable soil.

In conclusion, this is the first study that provides information on the whole genome sequence of a novel biological control agent commercialized recently in Europe and US. Using this approach, we unraveled its taxonomy as a strain of *B. subtilis*, while, in addition, we identified in the genome of MBI 600 a series of several genes that may play a crucial role in plant growth promotion, root colonization ability and biological control of plant pathogens. However, further research is required to confirm, by chemical analytical methods, the production of metabolites encoded by these genes, that are implicated to growth promotion and/or biological control of pathogens. Furthermore, by taking advantage of a functional homologous recombination system we successfully obtained a *yfp*-labeled MBI 600 mutant enabling us to localize the growth patterns of bacterial cells on cucumber roots using confocal microscopy. By using this tool we showed that MBI 600 exhibits only epiphytic growth, while bacterial cells maybe persistent on the root surface at least 20 days post inoculation. *In vitro* measurements of antagonistic effects and pot experiments showed that MBI 600 can effectively control 2 major pathogens of cucumber, *P. aphanidermatum* and *Forc* that is hard to be managed successfully with conventional chemicals. However, further research is required to optimize the use of MBI 600 under field/greenhouse conditions, while additional studies on the effect of MBI 600 against foliar pathogens of cucumber or against pathogens in other crops may contribute to the expansion of its use in agricultural crops.

## Data Availability Statement

The datasets presented in this study can be found in online repositories. The names of the repository/repositories and accession number(s) can be found in the article/Supplementary Material.

## Author Contributions

AS was the principal investigator, conceived and designed the experiments, and contributed to the writing of the manuscript. MN conducted the bioinformatic analysis. TM was responsible for confocal microscopy studies. GA conducted the bioinformatic analysis and wrote parts of the manuscript. MA-G and JC-A contributed to the transformation procedure of MBI 600. DR supervised the Bacillus transformation procedure and wrote the related part of the manuscript. GK supervised the study, conceived, and designed the experiments in collaboration with AS and wrote part of the manuscript. All authors read and approved the final manuscript.

## Conflict of Interest

The authors declare that the research was conducted in the absence of any commercial or financial relationships that could be construed as a potential conflict of interest.

## Supplementary Material

Supplementary Figure 1 A phylogenetic tree showing relationships between a few closely related plant-associated Bacillus species strains, including *B. subtillis*, *B. atrophaeus*, *B. amyloliquefaciens*, *B. velezensis*. *B. licheniformis*—B. paralicheniformis was used as the outlier.

Supplementary Figure 2 Growth promotion in 35 days-old cucumber plants treated with MBI 600, compared to non-treated control plants.

Supplementary Figure 3 Growth curves studies of yfp- and non-yfp-tagged bacterial cells of *Bacillus subtilis* MBI 600 strain.
