# Supplementary figures and images for: Whole Genome Sequencing and Root Colonization Studies Reveal Novel Insights in the Biocontrol Potential and Growth Promotion by Bacillus subtilis MBI 600 on Cucumber
Source: Front Microbiol. 2021 Jan 12;11:600393. doi: 10.3389/fmicb.2020.600393 (PMC7837180; doi:10.3389/fmicb.2020.600393)

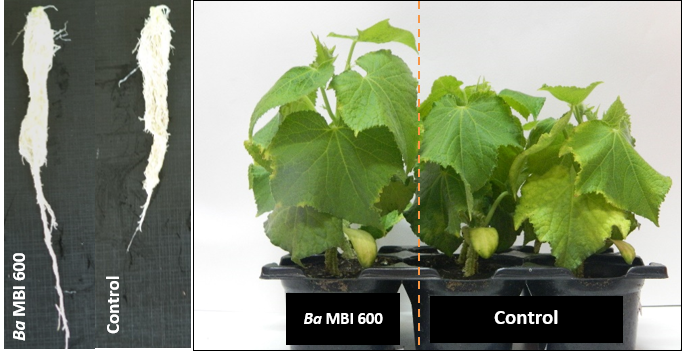

Supplement: Supplementary Figure 1 — A phylogenetic tree showing relationships between a few closely related plant-associated Bacillus species strains, including B. subtillis, B. atrophaeus, B. amyloliquefaciens, B. velezensis. B. licheniformis—B. paralicheniformis was used as the outlier. [file Image_1.PNG]

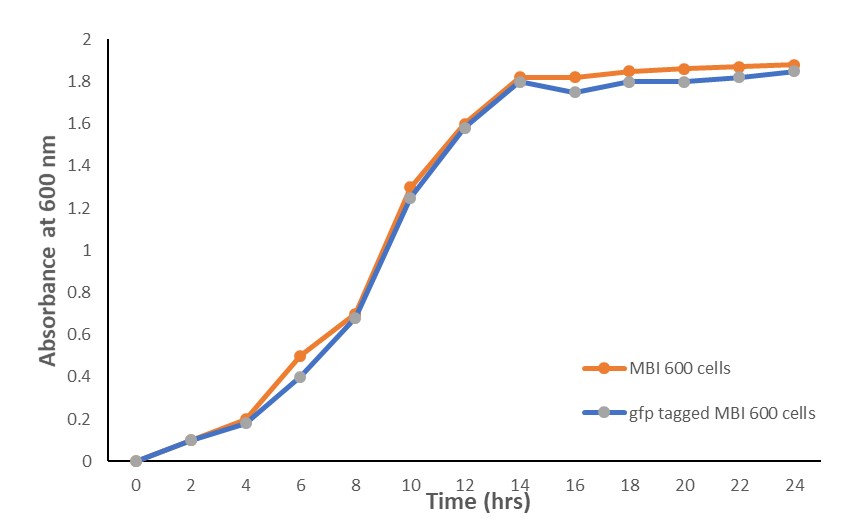

Supplement: Supplementary Figure 2 — Growth promotion in 35 days-old cucumber plants treated with MBI 600, compared to non-treated control plants. [file Image_2.JPEG]

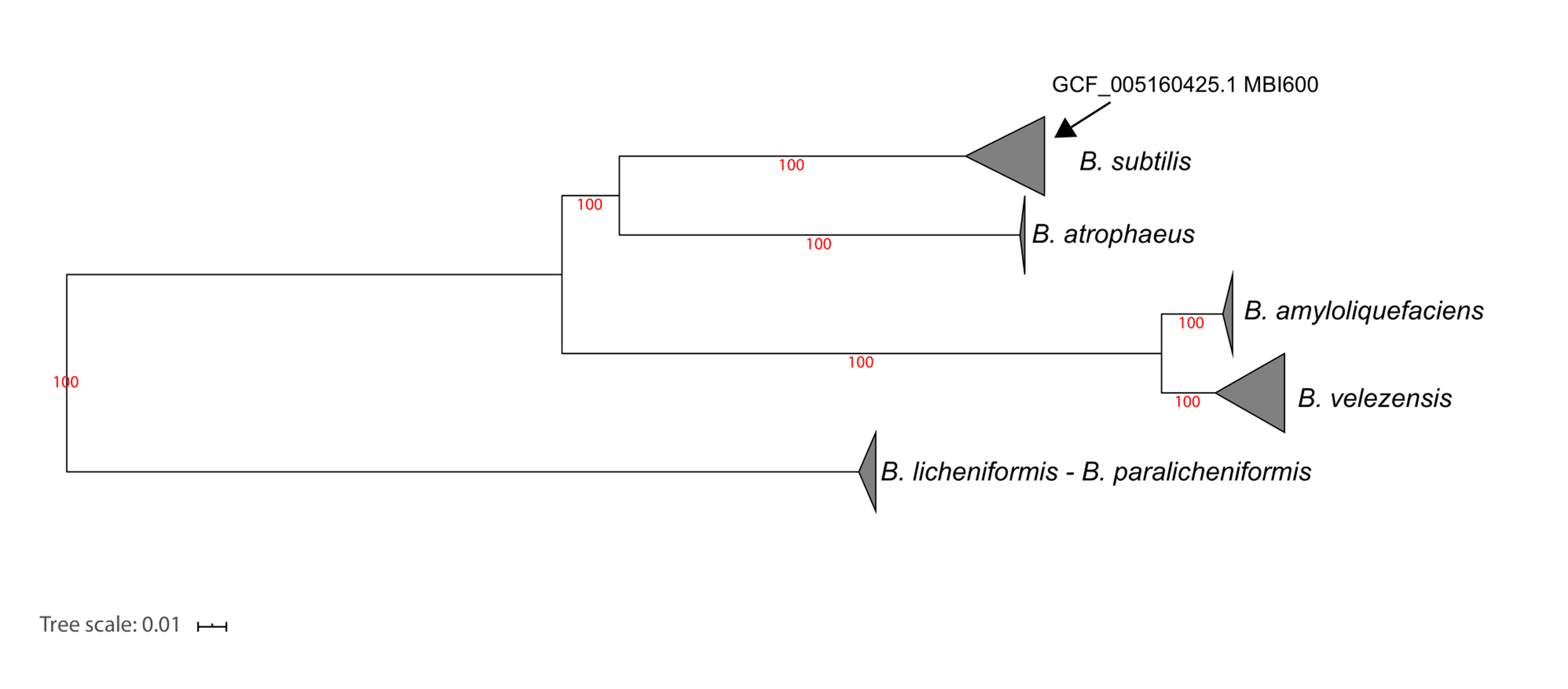

Supplement: Supplementary Figure 3 — Growth curves studies of yfp- and non-yfp-tagged bacterial cells of Bacillus subtilis MBI 600 strain. [file Image_3.TIFF]
